# Genetic characterization of hantaviruses isolated from rodents in the port cities of Heilongjiang, China, in 2014

**DOI:** 10.1186/s12917-016-0695-7

**Published:** 2016-04-02

**Authors:** Suya Cao, Jian Ma, Cheng Cheng, Wendong Ju, Yulong Wang

**Affiliations:** Department of Wildlife Medicine, Wildlife Resources Faculty, Northeast Forestry University, Harbin, 150040 China; State Key Laboratory of Veterinary Biotechnology, Harbin Veterinary Research Institute, the Chinese Academy of Agriculture Sciences, Harbin, 150001 China; Heilongjiang International Travel Healthcare Center, Harbin, 150001 China

**Keywords:** Hantavirus, SEOV, HTNV, Epidemiology, Heilongjiang

## Abstract

**Background:**

Hantavirus is a tripartite negative-sense RNA virus. It can infect humans through contaminated rodent excreta and causes two types of fatal human diseases: hemorrhagic fever with renal syndrome (HFRS) and hantavirus pulmonary syndrome (HPS). China exhibits the highest HFRS occurrence rate in the world, and the Heilongjiang area is one of the most severely infected regions.

**Results:**

To obtain additional insights into the genetic characteristics of hantaviruses in the port cities of the Heilongjiang area in China, a molecular epidemiological investigation of hantaviruses isolated from rodents was performed in 2014. A total of 649 rodents (11 murine species and 1 shrew species) were caught in 12 port cities in Heilongjiang. Among these rodents, the most common species was *A. agrarius*, and the second-most common was *R. norvegicus*. A viral gene PCR assay revealed the presence of two specific genotypes of hantavirus, referred to as Hantaan virus (HTNV) and Seoul virus (SEOV), and the positive SEOV infection rate was higher than that for HTNV. A genetic analysis based on partial M segment sequences indicated that all of the isolates belonging to SEOV could be assigned to two genetic lineages, whereas the isolate belonging to HTNV could be assigned to only one genetic lineage.

**Conclusions:**

These results suggested that HTNV and SEOV are circulating in *A. agrarius* and *R. norvegicus* in the port cities in the area of Heilongjiang, but SEOV may be the dominant common hantavirus.

## Background

Hantaviruses, which belong to the Hantavirus genus in the *Bunyaviridae* family, are tripartite negative-sense RNA viruses. Hantaviruses possess a tripartite negative-sense RNA genome consisting of the following three segments: the large (L) segment encodes a viral RNA-dependent RNA polymerase; the medium-sized (M) segment encodes two viral glycoproteins (GPs, Gn and Gc); and the small (S) segment encodes the viral nucleocapsid protein (NP) [1–5]. The GPs along with the NP determine the virulence and pathogenicity of the hantavirus. Unlike other viruses of the Bunyaviridae family, hantaviruses are not transmitted by arthropods; rather, they infect people though the urine, saliva and feces excreted by rodent hosts, especially muroids [6, 7]. Hantaviruses only generate transient pathology in rodents, and they do not affect the life span and reproduction of their hosts. In contrast, they can cause two severe clinical manifestations in humans: hemorrhagic fever with renal syndrome (HFRS) in the old world and hantavirus cardiopulmonary syndrome (HPS) in the new world [8–12]. Previous studies have indicated that at least 40 species and 30 genotypes belonging to the hantavirus genus have been isolated worldwide [13].

Etiological studies have shown that HFRS that has spread around the world, resulting in the production of variant hantaviruses. In Asia and Europe, five types of hantaviruses can cause HFRS: Hantaan virus (HTNV), Seoul virus (SEOV), Dobrava virus (DOBV), Saaremaa virus (SAAV), and Puumala virus (PUUV) [14]. In the USA, the Sin Nombre virus (SNV) and the Andes virus (ANDV) are stable viruses that can cause HPS. Most HFRS cases occur in Europe and East Asia (Korea, China and the eastern part of Russia) [11, 15]. China is the country that is most seriously affected by hantavirus infection worldwide. Previous investigations have shown that HFRS-infected patients in East Asian countries, including China, Russia and Korea, account for at least 90 % of HFRS patients around the world. At least 100,000 cases of HFRS are reported annually in China, and more than 900 cases are reported in Korea and the eastern part of Russia [16, 17]. HFRS is caused by different types of hantaviruses in different countries, and phylogenetic analysis suggests that hantaviruses and rodent hosts have coevolved [3]. In China, there are two common types of hantavirus, HTNV and SEOV, which are reportedly carried by *A. agrarius* and *R. norvegicus,* respectively, and some hantaviruses isolated from *R. norvegicus* seemed to be HTNV [16, 18, 19].

HTNV and SEOV could cause serious public health problems in China, especially in the Heilongjiang area of China. Heilongjiang is located in northeastern China, and it is adjacent to northern Russia and the nearby Jilin area to the south. It was the first area in which the etiological agent of HFRS was isolated in China, [11] and the Heilongjiang area has remained a high-incidence region [20]. Previous studies have shown that SEOV and HTNV are circulating in the Heilongjiang area [21, 22].

The port of Heilongjiang serves as bridge between countries. A single commercial port is used for economic trade, the exchange of technology and culture, tourism, immigration and so on. Given that many anthropozoonoses are transmitted by vectors (material, people and animals), and these vectors might be introduced into China through ports, it is essential to investigate the molecular epidemiology of hantaviruses by monitoring the rodents that are the natural hosts of hantavirus in the port cities of the Heilongjiang area. In this study, 12 port cities have been selected as trapping sites for our hantavirus investigation. We captured 649 rodents and characterized 29 hantaviruses isolated from various rodent species in the port cities. A phylogenetic analysis of the partial M segments indicated that 19 of 29 viruses belonged to SEOV, and 10 of 29 viruses belong to HTNV. Entire M segment of HTNV (TJ strain) which isolated from *R. norvegicus* have been sequenced and compared with the reference HTNVs. The results indicated that the spillover of HTNV has occurred under natural condition in port cities of Heilongjiang area.

## Methods

### Rodent collection

**Fig. 1:**
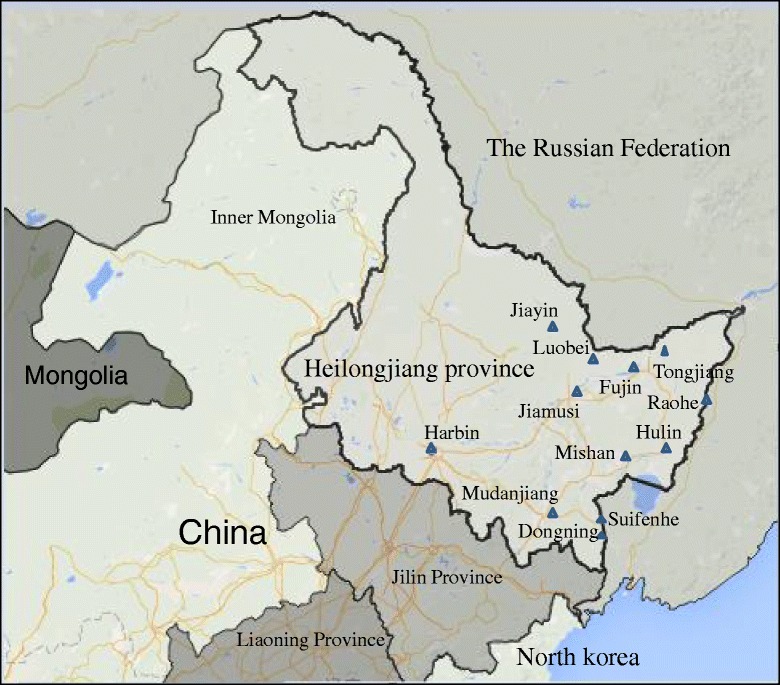
Map showing the trapping sites for rodents in the Heilongjiang area, China. Almost all of the port cities are located along the border with Russia, except for Harbin and Jiamusi. 48 rodents were captured in Luobei, 79 in Jiayin, 60 in Jiamusi, 22 in Dongning, 36 in Hulin, 27 in Mudanjiang, 102 in Mishan, 95 in Harbin, 29 in Fujin, 60 in Tongjiang, 22 in Suifenhe, and 69 in Raohe

In 2014, rodents were trapped with rat traps or baited cages in the 12 port cities of Luobei, Jiayin, Jiamusi, Dongning, Hulin, Mudanjiang, Mishan, Harbin, Fujin, Tongjiang, Suifenhe, and Raohe (Fig. 1). The trap sites included tussocks, brushwood, residential areas, canals and fields, among other areas. All of the trapped rodents were identified by zootaxy experts, and they were dissected as soon as they were authenticated. Lung tissues were collected from the captured rodents and transported to the HEILONGJIANG ENTRY-EXIT INSPECTION AND QUARANTINE BUREAU at –80 °C and stored in a liquid nitrogen tank until further processing. All procedures used in this study were approved by the Institutional Animal Care and Use Committee of Heilongjiang International Travel Healthcare Center (Harbin, China) (XD-25; 12 April 2014).

### RNA extraction and RT-PCR

The RNAprep Pure Tissue Kit (TIANGEN, Beijing, China) was used to extract total RNA from lung tissues, after the lung tissues were ground into a homogeneous mixture with a Tissue Lyser (Qiagen, Hilton, Germany). Purified total RNA was reverse-transcribed using the Superscript First-Strand Synthesis System (Invitrogen, Beijing, China) and random primers (Invitrogen, Beijing, China) to obtain cDNA. The HTNV and SEOV genotypes were amplified via nested PCR. The initial round of PCR product amplification employed the primer pairs HTm1F (5′-AAA GTA GGT GTT AYA TCY TTA CAA TGT GG-3′) and HTm1R (5′-GTA CAT CCT GTR CCT ACC CC-3′), and the second round of PCR production used the primer pairs HTm2F (5′-GAA TCG ATA CTG TGG GCT GCA AGT GC-3′)/HTm2R (5′-GGA TTA GAA CCC CAG CTC GTC TC-3′) and SEm2F (5′-GTG GAC TCT TCT TCT CAT TAT T-3′)/SEm2R (5′-TGG GCA ATC TGG GGG GTT GCA TG-3′). The initial round of PCR cycling consisted of an initial denaturation at 95 °C for 5 min, followed by 35 cycles of denaturation at 94 °C for 30 s, annealing at 52 °C for 30 s, and elongation at 72 °C for 30 s, with a final extension at 72 °C for 10 min. The second round of PCR cycling consisted of an initial denaturation at 95 °C for 5 min, followed by 30 cycles of denaturation at 94 °C for 30 s, annealing at 55 °C for 30 s, and elongation at 72 °C for 30 s, with a final extension at 72 °C for 10 min. The amplified products were approximately 330 bps.

For avoiding possible false positive results caused by RT-PCR assays, a commercial hantavirus detection kit (Hantavirus qPCR assay kit, Huiruibio, China) was also used to confirm the positive results determined by RT-PCR.

The whole M gene of HTNV isolate (TJ) was further amplified by RT-PCR method as following: The 5′ fragement of M gene was amplified by the primer pairs as : HM1F (5′- CAA CAT TAT ATA TGA TTG TAC CGA T -3′)/HM1R (5′- TGA ACC TGT GAG TTA CCT GGC ATA C -3′), and the 3′ fragement of M gene was amplified by the primer pairs as : HM2F (5′- AGA TGT TAT ATC TTT ACA ATG TGG G -3′)/HM2R (5′- CAC TCT CTG CAC CAT AAC AGA TAG C -3′). The PCR cycling consisted of an initial denaturation at 95 °C for 5 min, followed by 35 cycles of denaturation at 94 °C for 30 s, annealing at 55 °C for 30 s, and elongation at 72 °C for 90 s, with a final extension at 72 °C for 10 min. The sequence of entire M gene with approximately 1500 bps was obtained by assembling the above two fragements sequences by using DNAStar software.

### Phylogenetic analysis of M gene sequences

The PCR products were purified with the EZNA TM Gel Extraction Kit (OMEGA, USA) according to the manufacturer’s instructions and sequenced with the same primers used for PCR amplification. The nucleic acid sequences of the hantaviruses and those downloaded from GenBank were edited and analyzed with the DNASTAR program (DNASTAR, Madison, WI, USA). The identities of the isolated and downloaded hantavirus sequence were also calculated with the DNASTAR program.

The Seqman program was used to edit the nucleic acid sequences. The MEGA 4.0 program was employed to generate a viral phylogenetic tree via the neighbor-joining (N J) method. The alignment program of DNAStar software and Gendoc software were used to complete the alignment analyses of amino acid sequence of M segment

## Results

### Descriptions of the sampled rodents

In 2014, 649 rodents were captured from the 12 port cities in the Heilongjiang area of China. The rodents included 282 specimens of *A. agrarius*, 180 *R. norvegicus*, 15 *Clethrionomys rufocanus Sundevall*, 32 *A. peninsulae*, 20 *Clethrionomys rutilus Pallas*, 9 *Mus musculus*, 43 *Microtus fortis Buchner*, 3 *R. rattus*, 14 *E. sibiricus*, 44 *C. triton*, 3 *S. dauricus*, and 4 *Sorex araneus Linnaeus*. The Heilongjiang area harbors a diversity of species, with *A. agrarius* and *R. norvegicus* representing the dominant species. Nested PCR targeting partial M segment sequences could be applied to screen the infected rodents in all samples. The results showed that the infection rate of *A. agrarius* was 4.9 %, and the infection rate of *R. norvegicus* was 8.3 % (Table 1).

**Table 1:** Detection of hantavirus in rodent species captured at trap sites in various locations

Location	Species																							
	*A. agrarius*		*R. norvegicus*		*Clethrionomys rufocanus* Sundevall		*A. peninsulae*		*Clethrionomys rutilus* Pallas		*Mus musculus*		*Microtus fortis* Buchner		*R. rattus*		*E. sibiricus*		*C. triton*		*S. dauricus*		*Sorex araneus* Linnaeus	
Luobei	0/5^(a)^	0^(b)^	0/3	0	-^(c)^	-	0/2	0	0/11	0	-	-	0/25	0	-	-	0/2	0	-	-	-	-	-	-
Jiayin	1/43	2.3	0/14	0	0/5	0	-	-	0/1	0	0/5	0	0/2	0	0/1	0	0/6	0	0/2	0	-	-	-	-
Jiamusi	0/15	0	4/43	9.3	-	-	-	-	-	-	0/1	0	-	-	-	-	-	-	-	-	-	-	0/1	0
Dongning	0/5	0	5/14	35.7	-	-	-	-	-	-	-	-	-	-	-	-	0/3	0	-	-	-	-	-	-
Hulin	1/21	4.8	0/4	0	0/7	0	0/1	0	0/3	0	-	-	-	-	-	-	-	-	-	-	-	-	-	-
Mudanjiang	0/21	0	2/6	33.3	-	-	-	-	-	-	-	-	-	-	-	-	-	-	-	-	-	-	-	-
Mishan	1/51	1.9	0/25	0	0/3	0	0/17	0	-	-	0/1	0	-	-	-	-	0/2	0	0/3	0	-	-	-	-
Harbin	4/36	11	0/18	0	-	-	-	-	-	-	-	-	-	-	0/2	0	-	-	0/36	0	0/3	0	-	-
Fujin	1/11	9.1	0/15	0	-	-	-	-	-	-	-	-	0/3	0	-	-	-	-	-	-	-	-	-	-
Tongjiang	3/46	6.5	1/11	9.1	-	-	-	-	-	-	-	-	0/1	0	-	-	-	-	-	-	-	-	0/2	0
Suifenhe	0/5	0	0/5	0	-	-	0/7	0	-	-	-	-	-	-	-	-	0/1	0	0/3	0	-	-	0/1	0
Raohe	3/23	13	3/22	13.6	-	-	0/5	0	0/5	0	0/2	0	0/12	0	-	-	-	-	-	-	-	-	-	-
Total	14/282	4.9	15/180	8.3	0/15	0	0/32	0	0/20	0	0/9	0	0/43	0	0/3	0	0/14	0	0/44	0	0/3	0	0/4	0

^(a)^Number of positive samples/Number of tested samples

^(b)^Positive rate (%)

^(c)^No Samples

**Table 2:** The information of the Hantavirus islalated in this Study

Genotype	Isolate	Geographic Location	Accession No.
HTNV	CJAp93	Jilin	EF208930
HTNV	CGHu1	Guizhou	EU092222
HTNV	Lee	South Korea	D00377
HTNV	76-118	South Korea	Y00386
HTNV	LR1	China	AF288293
HTNV	S85-46	China	AF288658
HTNV	84FLi	Sanxi	AF366569
HTNV	SN7	China	AF288656
HTNV	N8	-	EF077656
HTNV	Z10	Zhejiang	NC_006437
HTNV	ZLS6-11	Zhejiang	FJ753397
HTNV	A6	China	AF288645
HTNV	HV114	Hubei	L08753
HTNV	h5	Helongjiang	L08753
HTNV	a16	Sanxi	AF288645
HTNV	AH09	China	AF285265
HTNV	jilinap06	Jilin	EF371454
HTNV	Q32	Guizhou	DQ371905
HTNV	CGRni1	Guizhou	EU363815
HTNV	TJJ16	Tianjin	EU074672
HTNV	CGAa4MP9	Guizhou	EF990929
HTNV	CGAa1015	Guizhou	EF990926
HTNV	NC167	Anhui	DQ989237
HTNV	Bao14	Helongjiang	AB127995
HTNV	TJF3	Tongjiang	KT885159
HTNV	TJF2	Tongjiang	KT885160
HTNV	TJF1	Tongjiang	KT885161
HTNV	HRH14	Raohe	KT885162
HTNV	HRH13	Raohe	KT885163
HTNV	HMS1	Mishan	KT885164
HTNV	HMDJ6	Mudanjiang	KT885165
HTNV	HJY10	Jiayin	KT885166
HTNV	HJI14	Harbin	KT885167
HTNV	HHL11	Hulin	KT885168
SEOV	hebei4	Hebei	AB027089
SEOV	Z37	Zhejiang	AF190119
SEOV	ZT71	Zhejiang	EF117248
SEOV	Sapporo	Sapporo	M34882
SEOV	80-39	South Korea	S47716
SEOV	hubei-1	Hubei	S72343
SEOV	Tchoupitoulas	-	U00473
SEOV	Vietnam5CSG	Vietnam	AB618130
SEOV	guang199	-	AB027086
SEOV	Houston	UK	U00465
SEOV	HN71-L	Hainan	AB027084
SEOV	K24-V2	-	AF288654
SEOV	L99	Jiangxi	AF288298
SEOV	HB55	Henan	AF035832
SEOV	R22	Henan	AF035834
SEOV	Brazil	Brazil	U00460
SEOV	humber	UK	JX879768
SEOV	IR461	UK	AF458104
SEOV	Gou3	zhejiang	AF145977
SEOV	SDN2	Dongning	KT885169
SEOV	SDN8	Dongning	KT885170
SEOV	SDN10	Dongning	KT885171
SEOV	SDN12	Dongning	KT885172
SEOV	SDN13	Dongning	KT885173
SEOV	SDN19	Dongning	KT885174
SEOV	SJC12	Harbin	KT885175
SEOV	SJC20	Harbin	KT885176
SEOV	SJI1	Harbin	KT885177
SEOV	SJMS16	Jiamusi	KT885178
SEOV	SJMS19	Jiamusi	KT885179
SEOV	SJMS21	Jiamusi	KT885180
SEOV	SJMS30	Jiamusi	KT885181
SEOV	SMDJ24	Mudanjiang	KT885182
SEOV	SRH17	Raohe	KT885183
SEOV	SRH19	Raohe	KT885184
SEOV	SRH27	Raohe	KT885185
SEOV	SRHA1	Raohe	KT885186
SEOV	TJF11	Tongjiang	KT885187

### Genetic diversity of SEOV isolates

**Fig. 2:**
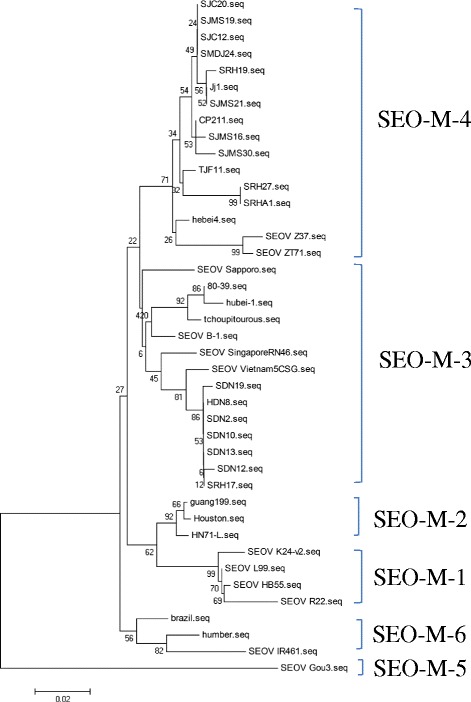
Phylogenetic tree of Seoul virus isolates based on the partial sequences of M segments. The tree was constructed by the neighbour-joining method (NJ) of Mega software. The HTNVs isolated from *R. norvegicus* or *A. agrarius* that were trapped in Harbin were designated SJC20, ji1 and SJC12; those iaolated from Jiamusi, SJMS19, SJMS21, SJMS16, and SJMS30; those isolated fromSuifenhe, SRH19, SRH27, SRH17 and SRHA1; that isolated from Mudanjiang, SMDJ24; that iaolated from Tongjiang, TJF11; and those isolated from Dongning, HDN8, SDN2, SDN10, SDN12 and SDN13. The GenBank accession numbers of the viruses isolated in this study and the reference HTNV stains were listed in Table 2 in detailedly

Phylogenetic tree was constructed with the partial M segment sequences of the viruses (Fig. 2). In the phylogenetic tree analyses of the SEOV isolates, the calculations showed that all of isolates formed six lineages. Lineages 1 to 4 were quite closely related to one another. All of the isolates in lineages 1, 2, and 3 came from China, while the viruses in lineage 4 were isolated from China and neighboring countries (Japan, Korea, Singapore and Vietnam) as well as the United States. In lineage 4, the isolates came from Jiamusi, Mudanjiang, Harbin and Raohe. The isolates show large sequence distances from strains Z37 and CP211, which were isolated from Zhejiang and Beijing. Lineage 3 consisted of the viruses isolated from Dongning and Raohe, and their virus sequences were very similar. All of the isolates were closely related to the Vietnam5CSG strain previously isolated from small mammals in Vietnam, which is far from the Heilongjiang area. Within lineage 1, strain L99 was isolated from the Jiangxi area, while strain K24-v2 came from the Zhejiang area, and these two areas share borders. The district from which strains R22 and HB55 were isolated, in the Henan area, is located far from the Zhejiang area. Lineage 5 consisted of Gou3, which was isolated from the Zhejiang area, and it appeared to be distinct from the other SEO viruses. Lineage 6 consisted of UK viruses, with the exception of one Brazil strain.

### Genetic diversity of HTNV

**Fig. 3:**
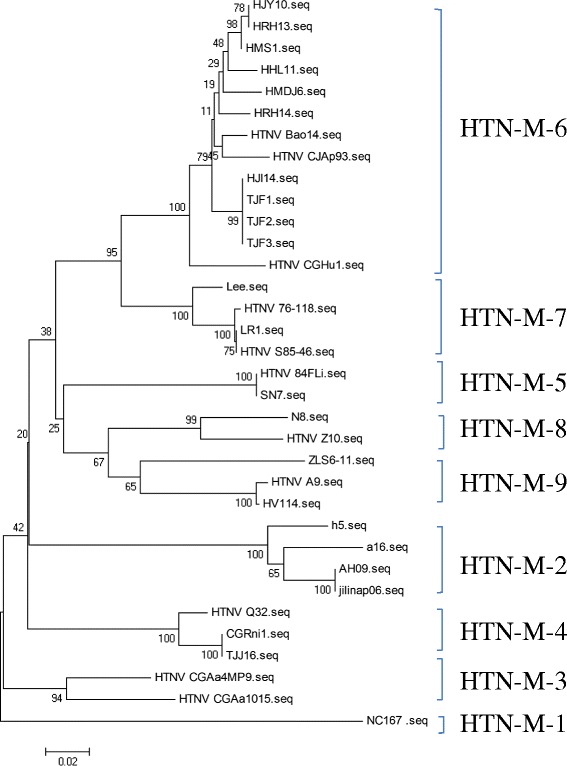
Phylogenetic tree (NJ) of hantaviruses based on partial M segment sequences of HTNVs from China. The HTNV that isolated from *R. norvegicus* or *A. agrarius* that were trapped in Harbin was designated HJI14; those isolated from Tongjiang, TJF1, TJF2 and TJF3; that isolated from Hulin, HHL11; that isolated from Mishan, HMS1; that isolated from Jiayin, HJY10; that isolated from Mudanjiang, HMDJ6; and those isolated from Raohe were designated HRH14 and HRH13. The GenBank accession numbers of the viruses isolated in this study and the referencial SEOV stains were showed in Table 2 in detailedly

The genetic diversity of the HTN isolates was lower than that of the SEO isolates based on the partial M segment sequences. The analysis of the obtained HTNV phylogenetic tree revealed nine lineages from all of the isolated genes (Fig. 3). The partial M segment sequences of HTNV that were isolated in this study belonged to only one lineage, lineage 6. This lineage has been designated the Far East (FE) lineage, which was identified in patients with serious HFRS. The viruses isolated from the port cities (Jiayin, Raohe, Mishan, Hulin, Mudanjiang and Harbin) in the Heilongjiang area appeared to be very closely related to one another. The Bao14 strain showed a similar sequence to the viruses isolated from the Heilongjiang area and the CJAp93 strain isolated from the Jilin area, which adjoins Heilongjiang. Some of the partial M segment sequences of the isolates from port cities were also closely related to HTNV strains that were isolated far from Heilongjiang, such as the CGHu1 strain originating from the Guizhou area. Lineage 7 consisted of several Korean viruses and shared a common evolutionary source with lineage 6. Lineage 1 consisted of the novel Hantaan virus type NC167, which was isolated from Anhui. It forms a separate branch in the phylogenetic tree.

### Phylogenetic analysis of HTN, SEO and prototype hantaviruses

**Fig. 4:**
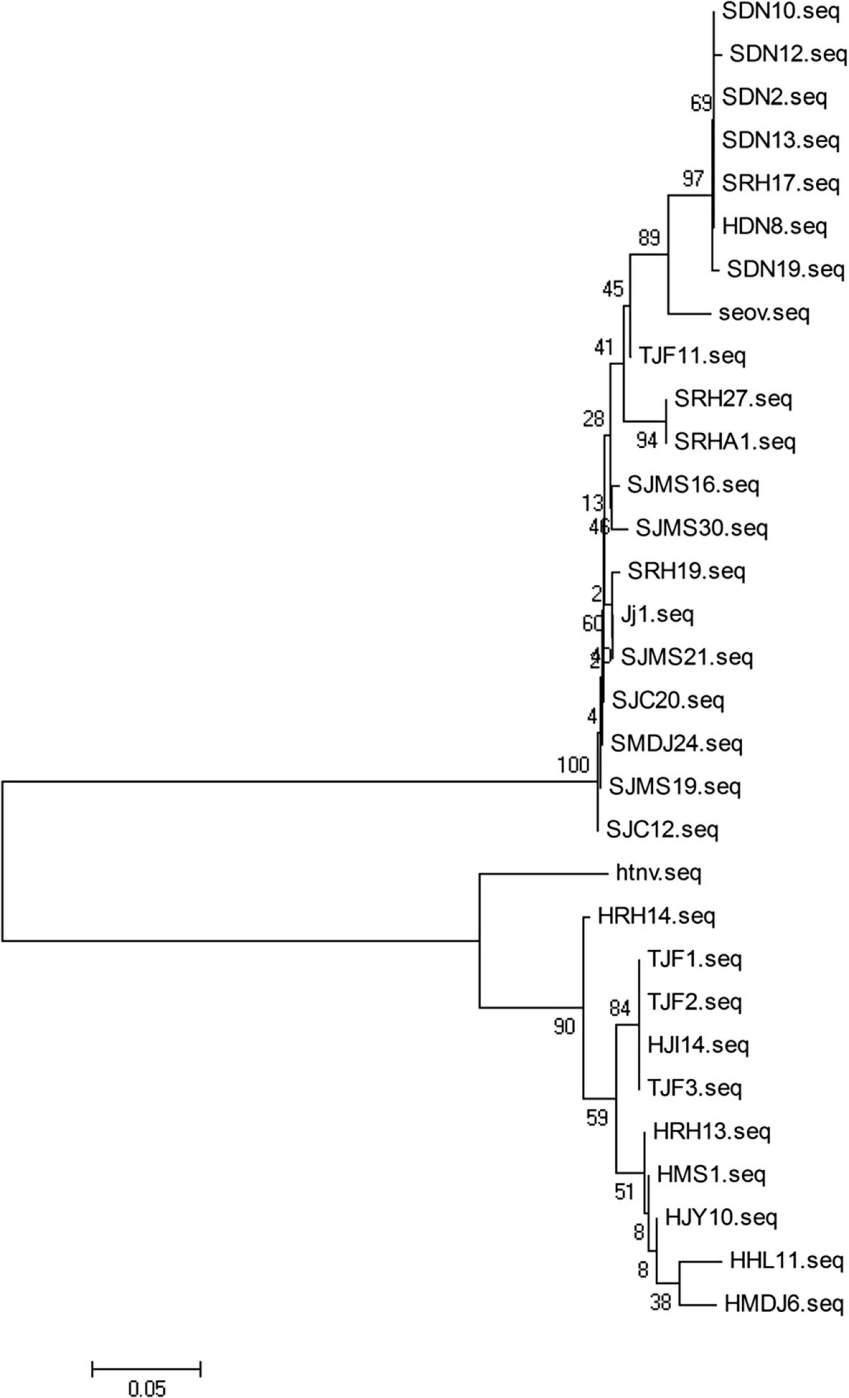
Phylogenetic tree (NJ) of hantaviruses based on partial M segment sequences of all Hantaviruses isolated in this study. The analysis was performed with MEGA software. The numbers at the nodes are bootstrap confidence levels for 1000 replicates. 19 partial M segment sequences of SEOV isolates and 10 partial M segment sequences of HTNV isolates were used to construct this phylogenetic tree with the reference sequences Hantaan 76-118 and Seoul 80-39. The GenBank accession numbers of the reference SEOV and HTNV genes were S47716 and Y00386 as showed in Table 2

To understand more about the HTNV and SEOV genetic variants circulating in rodent hosts in the port cities of the Heilongjiang area, we employed 19 partial M segment sequences of SEOV (KT885169-KY885187) and 10 partial M segment sequences of HTNV (KT885159-KT885168) to construct a phylogenetic tree with the reference sequences Hantaan 76-118 and Seoul 80-39 (Fig. 4). The phylogenetic analysis showed that the mutation rates of HTNV isolates were higher than the mutation rates of SEOV isolates, based on the reference hantavirus sequences.

### Comparison of amino acid sequences of M segment among HTNVs

**Fig. 5:**
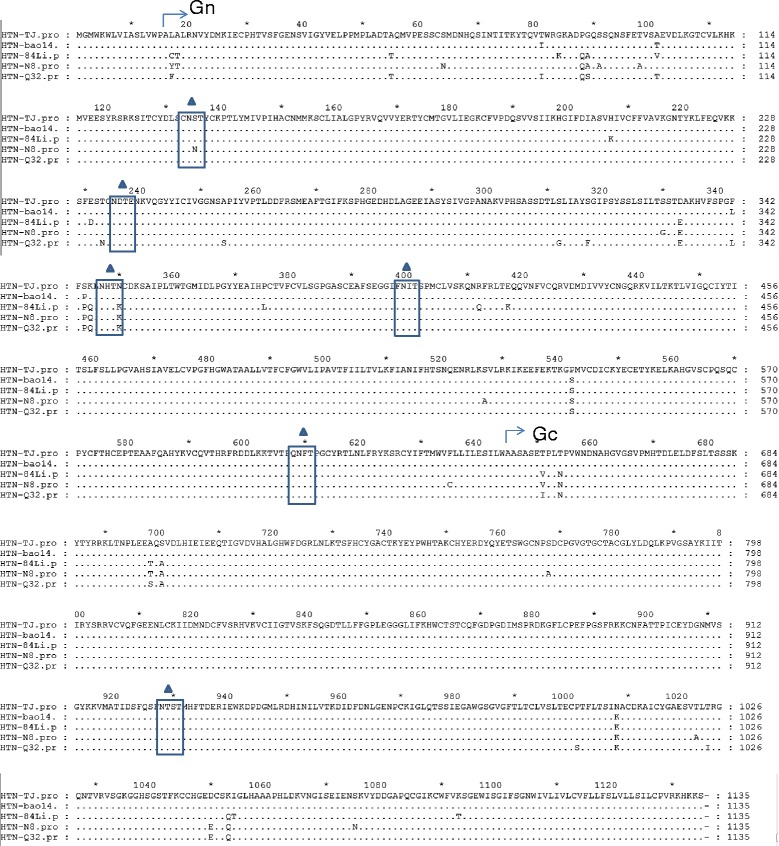
Multiple alignment of the deduced amino acid sequences based on M gene of hantaviruses isolate TJ and other reference stains. The amino acid differences between TJ isolate and other strains were shown. The function related N- glycosylation sites were framed and labled with filled triangles

The nucleotide sequence for M segment of TJ strain which isolated from *R. norvegicus* is 3408 nt, and a 1135 amino acid sequence could be translated by the nucleotide sequence. The alignment result in Fig. 5 showed that there was no deletion or insertion found in the deduced amino acid sequence based on M gene sequence of TJ strain. The identity of the deduced amino acid sequence between TJ strain and other HTNVs (Bao14, 84Li, N8 and Q32 strain) were more than 95 %. Among all the HTNVs, TJ strain showed a highest identity with Bao 14 strain (99.2 %), which was isolated from Heilongjiang too. The previous studies indicated that Five N- glycosylation sites in Gn (position 134, 235, 347, 399 and 609) and one N- glycosylation sites in Gc (position 928) were related with the function of M gene coding proteins [23]. However, all of the N- glycosylation sites were conserved among these different HTNVs (Fig. 5).

## Discussion

The Heilongjiang area is the most seriously affected endemic area of HFRS in China [13]. To gain a better understanding of the genetic characteristics of the hantaviruses present in the port cities in the Heilongjiang area, we collected 649 rodent specimens, and we detected hantaviruses in *A. agrarius* and *R. norvegicus*. The primary rodent hosts in the Heilongjiang area were *A. agrarius* and *R. norvegicus* for all of 2014. Among all of the evaluated rodents, 4.47 % were positive for hantavirus infection. In addition, we analyzed the genetic evolution of hantaviruses based on all of the positive isolates obtained during this study.

The rodent hosts in a specific area and the epidemic genotypes of hantaviruses exhibit a close relationship to epidemics of HFRS. This study showed that two hantaviruses (HTNV and SEOV) are circulating in the Heilongjiang frontier area of northeastern China at present. The epidemiological investigation showed that *A. agrarius* and *R. norvegicus* are the dominant rodent species and carriers of hantavirus in the suburban district and residential areas of the port cities in the Heilongjiang area (Table 1). Although the number of *R. norvegicus* specimens captured in this study was smaller than the number of *A. agrarius*, the *R. norvegicus* infection rate was higher than that of *A. agrarius. R. norvegicus* has become an advantageous rodent host for hantavirus, and the SEOV genotype carried by *R. norvegicus* has become the primary genotype of the virus. These results showed that HFRS occurring in the Heilongjiang frontier area might be caused primarily by SEOV. However, the possibility that other hantaviruses also play important roles in causing HFRS in this area could not be eliminated. Therefore, further studies will be necessary to determine whether other viruses are present in the Heilongjiang frontier area, and clinical samples will be needed to clarify the true etiological agents of HFRS in these regions.

Molecular epidemiological analysis has indicated that the SEOVs obtained in this study can be divided into six lineages in phylogenetic trees based on partial M segment sequences [24, 25]. Previous studies showed that lineages 1, 3 and 4 were present in northeastern China [26, 27]. However, we only isolated the viruses belonging to lineages 3 and 4 in the Heilongjiang frontier area in this study. Notably, these two virus lineages are distributed widely in China, especially in northeastern China and the middle and lower reaches of the Yangtze river [26, 28, 29]. Lineage 4 includes strains from Jilin, Zhejiang and Jiangxi, while lineage 3 includes strains from Japan, Vietnam and South Korea. We surmised that hantaviruses have spread around the world along with their primary natural reservoir, harbored by *R. norvegicus*. Moreover, according to Fig. 2, we observed that lineages 1— 4 exhibit a very close evolutionary relationship to lineage 6. These results indicated that most of the SEOVs isolated from around the world might have evolved from a single ancestor. However, a few variants, such as Gou3, which was isolated from Zhejiang and belongs to lineage 5, show a high degree of variability from most of the other SEOVs, which may suggest the evolution of an ancient hantavirus.

The first time that HTNV was reported was in Korea, and it was carried by *A. agrarius* [30]. Phylogenetic analysis showed that HTNV can be divided into nine lineages [25]. Previous work demonstrated the presence of lineage 6 in Heilongjiang Province, and lineage 7, which originated from Korea, is found widely throughout China [29, 31]. Several of the HTNVs that we detected are similar to the representative strain Bao14. Strains Bao14 and CJAp93, which belong to lineage 6 (lineage FE) and were isolated from Heilongjiang and Jilin, respectively, are the main pathogens responsible for HFRS in the northeast region of China. This finding may indicate that this HTNV variant is one of the pathogens associated with HFRS in northeastern China at present. However, we have not found any isolates that are similar to strain 76-118, which belongs to lineage 7 [31]. Previous studies have indicated that each dominant hantavirus genotype is associated with a specific rodent host, as HTNV is carried by *A. agrarius*, whereas the virus isolated from *R. norvegicus* has been identified as SEOV [32]. However, we isolated HTNVs from *R. norvegicus* in this study. This finding may be explained as a spillover infection, though we have not identified spillover of SEOV from *R. norvegicus* to *A. agrarius*. Furthermore, The entire M segment sequence of HTNV isolate (TJ strain) shared high identity with Bao 14 strain (99.2 %). It indicated that both TJ and Bao14 iaolates may evolve from the same ancestry. And we predicted that the transfer of HTNV infection host might be the approach employed by hantavirus to adapt to environmental change. In addition, all of the isolates (HTNVs and SEOVs) obtained in the present study belong to known lineages, and no new lineage of isolates has emerged in the Heilongjiang area.

## Conclusions

In this study, we found that HTNV and SEOV are circulating in the Heilongjiang frontier area and that SEOVs are the dominant common strains detected in rodent hosts. The HTNV isolates belong to lineage 6, and the SEOV isolates belong to lineages 3 and 4. All of the lineages are common in the northeast region of China. Although we have not identified a new lineage of hantavirus, regular epidemiological surveillance of local murine colonies is necessary and should be performed intensively.

## Abbreviations

ANDV: Andes virus
DOBV: Dobrava virus
HFRS: hemorrhagic fever with renal syndrome
HPS: hantavirus cardiopulmonary syndrome
HTNV: Hantaan virus
PUUV: Puumala virus
SAAV: Saaremaa virus
SEOV: Seoul virus
SNV: Sin Nombre virus
